# PTEN is recognized as a prognostic-related biomarker and inhibits proliferation and invasiveness of skull base chordoma cells

**DOI:** 10.3389/fsurg.2022.1011845

**Published:** 2022-09-23

**Authors:** Kaibing Tian, Junpeng Ma, Ke Wang, Da Li, Junting Zhang, Liang Wang, Zhen Wu

**Affiliations:** Department of Neurosurgery, Beijing Tiantan Hospital, Capital Medical University, Beijing, China

**Keywords:** Pten, skull base, chordoma, progression, invasion, p-AKT, p-mTOR

## Abstract

**Objective:**

This work aimed to examine the function of phosphatase and tensin homologue deleted on chromosome 10 (PTEN) in skull base chordoma (SBC) at the clinical and cellular levels.

**Methods:**

Totally 65 paraffin-embedded and 86 frozen specimens from 96 patients administered surgery were analyzed. Immunohistochemical staining and quantitative real-time polymerase chain reaction were performed, and the associations of PTEN expression with clinical features were assessed. At the cellular level, PTEN was knocked down by the siRNA approach in the UCH-1 cell line, and cell proliferation and invasion were detected by the CCK-8 and migration assays, respectively.

**Results:**

At the protein level, PTEN expression was increased in non-bone-invasive tumor samples in comparison with bone-invasive specimens (*p* = 0.025), and elevated in soft SBCs in comparison with hard tumors (*p* = 0.017). Increased PTEN protein expression was associated with decreased risk of tumor progression (*p* = 0.002; hazard ratio = 0.981, 95% confidence interval: 0.969–0.993). At the gene expression level, the cut-off value was set at 10.5 after ROC curve analysis, and SBC specimens were divided into two groups: PTEN high group, ΔCt value below 10.5; PTEN low group, ΔCt value above 10.5. In multivariate regression analysis of PFS, the risk of tumor progression was increased in PTEN low group tumors in comparison with PTEN high group SBCs (*p* = 0.006). In the CCK-8 assay, in comparison with control cells, PTEN knockdown cells had increased absorbance, suggesting elevated cell proliferation rate. In the invasion assay, the number of tumor cells penetrating into the lower chamber was significantly increased in the PTEN knockdown group compared with control cells.

**Conclusions:**

Decreased PTEN expression in SBC, at the protein and gene levels, is associated with reduced PFS. PTEN knockdown in chordoma cells led to enhanced proliferation and invasiveness.

## Introduction

Chordoma represents a low-grade malignancy originating from notochord remnants (1, 2) that mostly occurs in the sacrococcygeal (29.2%) and skull base regions (32%–42%) (1, 3, 4). This malignant disease affects 0.08–0.089/100,000 individuals, with a male predominance (morbidity rates of 0.1–0.16/100,000 and 0.06–0.066/100,000 in men and women, respectively) (4). Chordoma tumors can affect neighboring soft tissues and destroy surrounding bones. Skull base chordoma (SBC) is generally near critical blood vessels, cranial nerves and other major structures, and SBC patients often exhibit symptoms of cranial nerve dysfunction, including headache, paralysis, diplopia, decreased visual acuity, visual field defect, dysphagia, facial paralysis and numbness. Entire resection of cranial chordoma is hard to achieve and may result in serious complications, while chemotherapy generally has low efficacy. Currently applied treatments comprise radical resection and postsurgical radiation therapy, resulting in median patient survival in SBC of 151 months (5, 6). Postsurgical tumor progression represents an important challenge faced by patients as well as neurosurgeons. In recent years, despite revolutionizing advances in SBC treatment, the disease remains poorly understood at the molecular level (3, 7, 8).

Previously, brachyury and additional biomarkers were detected in chordoma (9, 10); however, molecular markers related to tumor features are rare. Phosphatase and tensin homologue deleted on chromosome 10 (PTEN) represents an important tumor suppressor protein that is poorly expressed in many malignant tumors (11–13). We recently demonstrated PTEN is involved in tumor invasion (14). The present study aims to further examine the function of PTEN in SBC at the clinical and cellular levels.

## Materials and methods

### Overview

Tumor specimens were obtained from SBC cases surgically treated between January 2008 and November 2015, and diagnosed as chordoma. Cases with paraffin-embedded samples assessable by immunohistochemistry or frozen samples usable for mRNA extraction were included. Exclusion criteria were: clinical data unavailability; other tumors or genetic disorders; other serious diseases that could affect prognosis. This study conformed to the Committee on Publication Ethics (COPE) and the International Committee of Medical Journal Editors (ICMJE) recommendations for ethics and had approval from Beijing Tiantan Hospital's ethics committee. Each patient provided signed informed consent. Data collection and analysis were performed in a blinded fashion.

### Clinical data

Demographic data and treatment history, including postsurgical radiation therapy, were obtained from medical records. Presurgical Karnofsky Performance Status (KPS) scores, surgical methods, blood supply and tumor texture were retrieved from the surgical records. Tumor blood supply was either abundant or poor, based on which the cases were categorized. Tumor texture was recorded as soft or hard (tumors with both soft and hard constituents were considered hard tumors). Follow-up was mostly performed on an outpatient basis, and phone calls were utilized for those unable to visit the hospital. Tumor progression was considered for recurrence or regrowth of the residual tumor.

**Figure 1:**
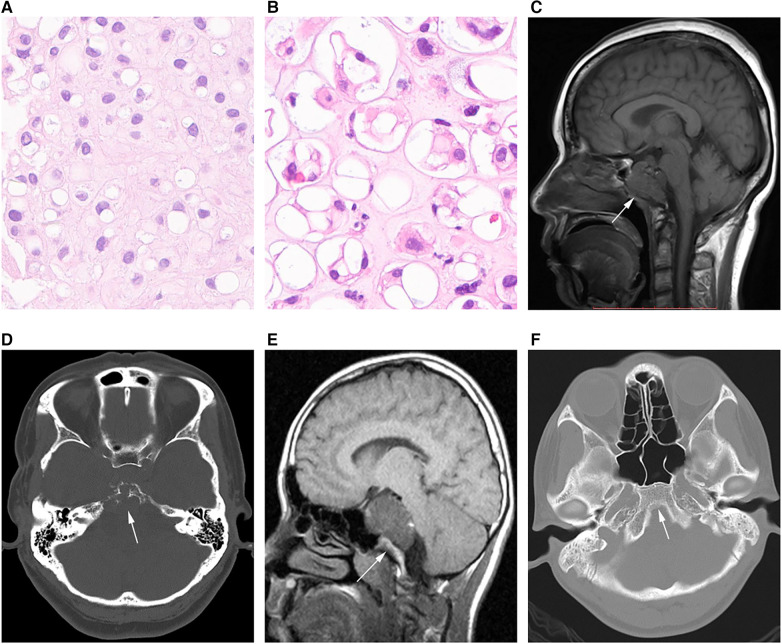
Different chordoma types. (**A**) Pathological image of classical chordoma showing many large cells with clear to eosinophilic cytoplasm; physalides are common in tumor cells. (**B**) Image of chondroid chordoma showing structures similar to the cartilage matrix between tumor cells. (**C,D**) MRI and CT scans in an SBC case showing that the tumor grows with the invasion of the whole clival bone (arrow). (**E,F**) CT image in another SBC case, with no bone invasion (arrow).

Histological analysis was performed by 2 or more pathologists with >10 years of experience in chordoma assessment. The tumors were grouped into conventional, chondroid, poorly differentiated and dedifferentiated types, as proposed by the International Agency for Research on Cancer (15). No poorly differentiated and dedifferentiated tumors were detected in the present analysis, which only comprised conventional (Figure 1A) and chondroid (Figure 1B) tumors.

Magnetic resonance (MR) imaging scans were assessed by two experienced radiologists with the Picture Archiving and Communication System. Tumor diameter was measured separately in three mutually perpendicular dimensions, with the greatest value considered the “diameter”. Invasiveness was considered for bone (Figures 1C,D) or non-bone (Figures 1E,F) invasive tumors. SBC stages were defined based on the presence of dura erosion. Tumor lobulation was reflected by unevenly lobulated borders, and the lobulation condition was recorded as with or without lobulation. The extent of resection was determined by postoperative MR imaging findings. Resection was classified as follows: (1) gross total resection, total resection of the visible tumor, with the affected bone ground for normal bone tissue exposure, and no tumor detected by postoperative MR imaging; (2) near total resection, total resection of any visible tumor with >90% of the tumor removed based on postsurgical MR imaging; (3) subtotal resection, 70%–90% of the tumor removed based on postsurgical MR imaging; (4) a partial resection and biopsy, <70% of the tumor removed. For simplification, the gross total resection and near total resection groups were considered aggressive resection (>90%), while subtotal resection, partial resection and biopsy were combined as non-aggressive resection (≤90%).

### Immunohistochemistry

Tumor specimens underwent formalin fixation in the 30 min following extraction from patients, paraffin-embedding, sectioning at and 5-µm. Then, the sections were dewaxed and hydrated. After antigen retrieval, the specimens underwent incubation with endogenous peroxidase and blocking. Next, sections were submitted to successive incubations with primary (1:400, Abcam, UK; 4°C overnight) and secondary (1:250, TransGen Biotech, China; 1 h at ambient). Finally, treatment with diaminobenzidine was followed by dehydration and mounting.

Five or more high-power fields were assessed per sample at 400×, and positivity rates for various samples were determined by two pathologists independently. Consensual discussions were applied to resolve any discrepancy.

### Quantitative real-time polymerase chain reaction (qRT-PCR)

**Figure 2:**
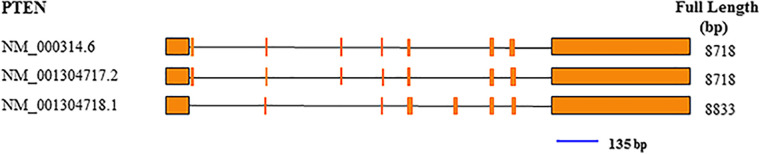
Exon composition of the PTEN gene, and position of the qRT-PCR amplicon (blue line). The amplicon was 135 bp.

Total RNA was extracted with TRIzol from tumor specimens snap frozen in liquid nitrogen in the initial 30 min following extraction from patients. In brief, 1 ml TRIzol (Thermo Fisher Scientific, Waltham, MA, USA) was mixed with 20 mg of tissue. PrimeScript RT reagent Kit with gDNA Eraser (Takara, Kusatsu, Shiga, Japan) was utilized for cDNA synthesis, as directed by the manufacturer. qRT-PCR was performed with TaqMan probes for PTEN (Figure 2) and GAPDH (Thermo Fisher Scientific, USA). Amplification was carried out in 10-µl reactions at 95°C (10 min), followed by 40 cycles of 95°C (15 s) and 60°C (60 s). The ΔCt values were used for further analysis.

In cellar level experiments, TRIzol was used for RNA extraction, followed by reverse transcription with Reverse Transcription Kit (Takara, Kyoto, Japan). Quantitative polymerase chain reaction (PCR) was carried out with real time-PCR Recording Kit (Takara) by the SYBR Green method. The primers were as follows: PTEN, forward 5′-GCC CTG TAC CAT CCC AAG TC-3′ and reverse 5′-GAT GCT GCC GGT AAA CTC CA-3′; GAPDH, forward 5′-GGA GCG AGA TCC CTC CAA AAT-3′ and reverse 5′-GGC TGT TGT CAT ACT TCT CAT GG-3′. Amplification was performed at 95°C (30 s), followed by 40 cycles of 95°C (5 s) and 60°C (30 s); or 95°C(15 s), 60°C (30 s) and 95°C (15 s). The 2^−ΔΔCT^ method was applied to examine transfection efficiency; transfection efficiency above 50% was considered to indicate a qualified transfection.

### Cell culture

The chordoma UCH-1 cell line was acquired from Chordoma Foundation (https://www.chordomafoundation.org). Pretreatment of culture plates/flasks was carried out with gelatin (Sigma-Aldrich, Inc., St. Louis, MO, USA) to increase cell adhesion. IMDM and RPMI 1,640 (Thermo Fisher Scientific) were used as the basic medium mixture (4:1), supplemented with nonessential amino acid solution (1×) and penicillin/streptomycin (1×) (Thermo Fisher Scientific) and 10% fetal bovine serum (Hyclone, Logan, UT, USA). This cell line was subcultured every three days.

### Cell proliferation assay

Cell transfection was performed by applying short interfering RNA (siRNA), the target siRNAs (Gima Genetics, Suzhou, China) were designed as follows: 5′-UGA ACC UGA UCA UUA UAG A-3′; the negative control siRNA was 5′-ATC TAG ATT AAC GAC ATT G-3′. SiRNA MATE^TM^ was utilized as the transfection reagent (Gima Genetics), and tumor cells were grown in 6-well plates in antibiotic-free medium to approximately 6  ×  10^4^/well; the medium was changed before transfection. The transfection medium comprised 6 pmol siRNA, 200 µl Opti-MEM medium, and 15 µl siRNA MATE™, and was added to tumor cells and incubated under routine conditions. After 6 h of transfection, the medium was changed, and transfection efficiency was examined after 48 h of incubation.

Cell Counting Kit-8 (CCK-8; Dojindo Molecular Technologies, Japan) was utilized for cell proliferation assessment. Cells were inoculated in a 96-well plate, and medium containing 10% CCK-8 was replaced after transfection for 24, 48 and 72 h, respectively. Absorbance at 450 nm was measured on a microtiter plate reader.

### Cell invasion assay

The 24-well transwell chambers (Corning, NY, USA) were applied for cell invasion experiments. The chambers were pretreated witt Matrigel (Corning), followed by addition of serum-free cell suspension and 20% serum-containing medium into the upper and lower chambers, respectively. Staining was performed after 12 h of incubation.

### Statistical analysis

SPSS (v. 20.0; IBM Corp., Armonk, NY, USA) was utilized for data analysis. Differences in PTEN expression between the SBC types were assessed by the t test or rank sum test. Receiver operating characteristic (ROC) curve analysis was carried out to set a cutoff value. The Kaplan-Meier (K-M) method was employed for examining survival and progression risks between different PTEN expression groups. In overall survival (OS) and progression-free survival (PFS) Cox regression models, each parameter was firstly included in univariable analysis; parameters with *p* < 0.05 were further assessed by multivariable analysis. *P* < 0.05 indicated statistical significance.

## Results

### Analysis of epidemiological data

Ninety-eight patients were included in this study. Only cases administered their first surgery in our hospital were assessed. Paraffin-embedded and frozen specimens were obtained from 65 to 86 cases, respectively. Both sample types were available for 53 cases. The patients were 11–64 years old (median, 39 ± 13.7 years), including 51 women and 47 men. Following the first surgical procedure in our hospital, 33 cases underwent another surgery for tumor progression, 2 had two further surgeries each, 3 had three further surgeries each, and 1 had four more surgeries for multiple postsurgical tumor progression events. Eighteen patients received postsurgical radiation therapy, 3 only had conventional radiation therapy, 12 only underwent stereotactic radiation therapy, 2 only had proton radiation therapy, and 1 had stereotactic and proton radiotherapies. In the present study, 26 cases died and 58 had tumor progression. Three-year OS was 70%. PFS rates at 3 and 5 years were 37% and 18%, respectively, and median PFS was 30.5 months. (More data in Table 1)

**Table 1:** Categorical variables for skull base chordoma patients.

	All patients	Patients with paraffin embedded samples	Patients with frozen samples
Sex			
Women	51	38	45
Men	47	27	41
Treatment history			
Yes	32	20	28
No	66	45	58
Invasion condition			
Bone invasion	88	58	77
Non-bone invasion	10	7	9
Stage			
Dura erosion	40	28	37
No dura erosion	58	37	49
Lobulation			
With lobulation	54	35	49
Without lobulation	44	30	37
PTEN expression			
High group			35
Low group			51
Texture			
Soft	42	27	38
Hard	56	38	48
Resection grade			
Aggressive resection	53	37	43
Non-aggressive resection	45	28	43
Pathology			
Conventional	66	48	59
Chondroid	32	17	27
Post radiotherapy			
Yes	18	11	17
No	80	54	69
Tumor progression			
Yes	58	40	54
No	40	25	32
Sum	98	65	86

Number of patients represent raw data.

### Association between PTEN protein expression and patients' clinical characteristics

**Figure 3:**
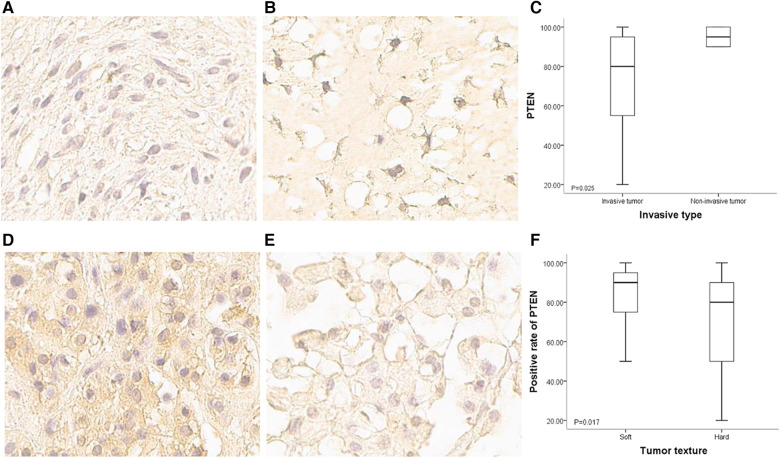
PTEN expression in SBCs of different invasiveness and texture conditions. (**A**) Immunohistochemical staining of PTEN for a bone invasive SBC (400×); staining signals were light, and mainly located in the cytoplasm and intercellular matrix. (**B**) Immunohistochemical staining of PTEN for a non-bone invasive SBC (400×); staining signals were strong, and the nucleus was positive. (**C**) Positivity rate for PTEN expression was higher in non-bone invasive tumors compared with bone invasive ones (*p* = 0.025). (**D**) Immunohistochemical staining of PTEN for a soft SBC (400×); staining signals were strong in the cytoplasm and intercellular matrix. (**E**) Immunohistochemical staining of PTEN for a hard SBC (400×); staining signals were weak, and the positivity rate was very low. (**F**) Positivity rate for PTEN was higher in soft tumors than in hard tumors (*p* = 0.017).

The 65 patients with paraffin-embedded specimens were 11 to 63 years old (median, 39 ± 14.1 years), including 38 women and 27 men. Age (*p* = 0.619) and sex (*p* = 0.420) were similar between this subgroup and the overall population. Table 1 lists all patient features. PTEN was mostly detected in the matrix, cytosol and nucleus. The positivity rates for all tumors were 20%–100% (median, 85%). PTEN protein expression significantly differed between SBC tumors of divergent invasive types or textures. PTEN was upregulated in non-bone-invasive tumor samples in comparison with bone-invasive tumor specimens (*p* = 0.025) (Figures 3A–C), and PTEN expression was increased in soft tumors in comparison with hard ones (*p* = 0.017) (Figures 3D–F). In univariable cox regression analysis of PFS, higher PTEN expression was correlated with reduced tumor progression risk (*p* = 0.002; hazard ratio [HR] = 0.981, 95% confidence interval [CI] 0.969–0.993).

### Association between PTEN gene expression and patients' clinical characteristics

**Figure 4:**
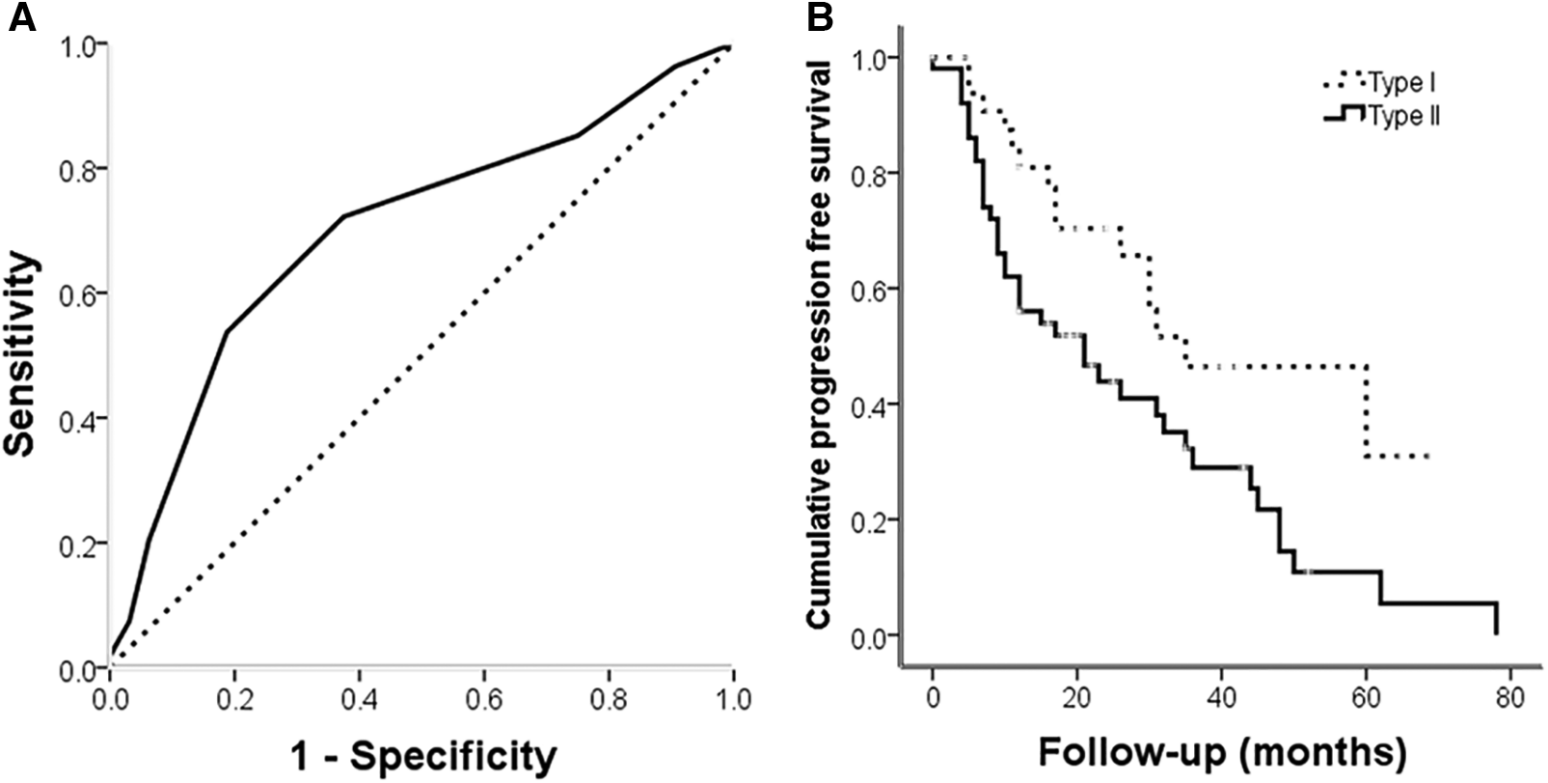
(**A**) ROC curve for PTEN expression at the gene level for tumor progression; the area under the curve was 0.699 (*p* = 0.002). (**B**) Kaplan-Meier analysis illustrating progression free survival for different PTEN expression groups. Progression free survival was longer in PTEN high expression group cases than in PTEN low expression group patients ((log rank = 6.1, *p* = 0.014).

The 86 cases with frozen specimens were 11–62 years old (median, 39 ± 13.8 years). In total, 45 women and 41 men were included. Age (*p* = 0.986) and sex (*p* = 0.846) were comparable between this subgroup and the overall population (Tables 1, 2). In univariable cox regression analysis of PFS, the ΔCt value of PTEN was a significant variable; a cut-off value was set at 10.5 after ROC curve analysis (Figure 4A), and SBCs were divided into two groups: PTEN high group, tumors with ΔCt value below 10.5 (higher PTEN expression); PTEN low group, ΔCt value above 10.5 (lower PTEN expression). After statistical analysis, patients' age, tumor diameter and presurgical KPS score were not significantly different between PTEN high and low groups, and no significant differences were found between PTEN high and low groups in patients with different sex, treatment history, pathological types, invasiveness status, stages and lobulation status (*p* < 0.05). In K-M curve analysis, PFS was increased in PTEN high group compared with PTEN low group (*p* = 0.014) (Figure 4B). After inclusion of presurgical KPS score, PTEN expression groups, patient sex and age, tumor diameter, treatment history, pathological type, invasiveness status, disease stage, lobulation status, resection grade and postsurgical radiation therapy in univariable cox regression analysis of PFS, treatment history, pathological type, PTEN expression groups, presurgical KPS score, tumor texture and resection grade were significantly associated with decreased PFS. The latter 5 parameters were further examined by multivariate regression analysis, which showed treatment history, pathological type and PTEN expression group independently predicted reduced PFS, and higher tumor progression risk was detected for PTEN low group compared with PTEN high group SBCs (*p* = 0.006), consistent with protein level data (Table 3).

**Table 2:** Continuous variables for skull base chordoma cases with frozen samples.

	Minimum	Maximum	Mean	Median	SD
Age (y)	11	62	36.6	39	13.8
Preoperative KPS	40	100	76.1	80	12.7
Diameter (mm)	15	85	44.6	44.9	14.1
ΔCt	8	16	11	11	1.6

Minimum and maximum values are based on raw data.

**Table 3:** Progression free survival analysis for skull base chordoma patients with frozen samples.

Parameter	Univariate analysis			Multivariate analysis		
	x ± sd	95% CI	*p*-value	HR	95% CI	*p*-value
Age			0.096			
Diameter			0.239			
Preoperative KPS			0.005*	0.992	0.967–1.017	0.508
Sex			0.173			
Men	36.7 ± 5.0	26.9–46.6				
Women	27.6 ± 3.4	21.0–34.3				
Treatment history			<0.001*	3.875	2.094–7.170	<0.001*
With	16.1 ± 3.2	9.9–22.3				
Without	39.0 ± 3.8	31.5–46.4				
Invasion condition			0.699			
Invasion	32.8 ± 3.3	26.2–39.3				
Non-invasion	25.1 ± 5.7	14.0–36.3				
Stage			0.717			
Dura erosion	33.1 ± 4.6	24.0–42.2				
No dura erosion	30.6 ± 3.7	23.3–37.9				
Lobulation condition			0.675			
No lobulation	33.0 ± 4.4	24.3–41.7				
Lobulation	31.2 ± 4.0	23.4–39.1				
Resection grade			0.025*	1.362	0.752–2.464	0.308
Aggressive resection	37.4 ± 3.8	30.0–45.0				
Non-aggressive resection	26.1 ± 4.2	17.9–34.4				
Texture			0.054			
Soft	39.6 ± 5.3	29.2–50.1				
Hard	26.7 ± 3.3	20.2–33.1				
Pathology			0.004*	0.485	0.248–0.948	0.034*
Conventional	26.3 ± 3.5	19.5–33.0				
Chondroid	43.5 ± 4.8	34.1–53.0				
Radiotherapy			0.902			
Without	32.0 ± 3.4	25.3–38.6				
With	30.4 ± 6.5	17.6–43.1				
PTEN expression			0.014*	2.425	1.282–4.588	0.006*
High	41.3 ± 4.9	31.8–50.8				
Low	26.4 ± 3.4	19.8–33.1				

HR, hazard ratio.

*p < 0.05.

### Effects of PTEN at the cellular level

**Figure 5:**
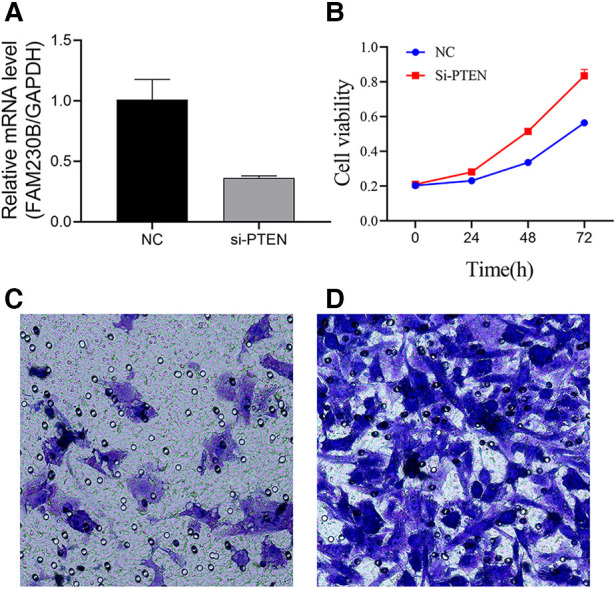
Effects of PTEN at the cellular level. (**A**) Histogram of PTEN knockdown efficiency. PTEN gene expression was significantly higher in the control group than in the knockdown group, with a knockdown efficiency of 64.1%. (**B**) Line graph of UCH-1 cell proliferation. Cell proliferation was higher after PTEN knockdown compared with control cells at 24 h, 48 h and 72 h, respectively. (**C,D**) Migrating cells observed at 200×. (**C**,**D**) are normal UCH-1 cells and PTEN knockdown cells, respectively. Cell migration was significantly increased in the PTEN knockdown group compared with the control group.

UCH-1 cells were transfected with siRNA to knockdown PTEN, and the knockdown rate was 64.1% after transfection (Figure 5A). Absorbance in cells treated with the CCK-8 reagent was measured by a microtiter plate reader. It was found that in comparison with control cells, absorbance in PTEN knockdown cells was increased, suggesting elevated cell proliferation rate (Figure 5B). In invasion assays, the number of tumor cells migrating into the lower chamber was significantly increased in the PTEN knockdown group in comparison with control cells, indicating an enhanced invasive ability for tumor cells in the PTEN knockdown group (Figures 5C,D).

## Discussion

In the present study, the protein and mRNA amounts of PTEN were assessed, and its functions and preliminary mechanism were further examined at the cellular level. The results indicated that decreased PTEN expression in SBCs had associations with tumor invasion, hard tumor texture and decreased PFS, and PTEN knockdown in tumor cells led to enhanced cell proliferation and invasion.

PTEN is encoded by a tumor suppressor gene commonly deleted in malignant diseases. PTEN protein is located in the cytoplasm and nucleus, and often secreted into the extracellular space (16). PTEN plays a role in dephosphorylating the cellular lipid signal phosphatidylinositol 3,4,5-trisphosphate, which acts antagonistically to phosphatidylinositol 3-kinase (PI3K) signaling to affect multiple cell processes, including growth, proliferation and polarization (17–18). In addition, PTEN inhibits other pathways, including the mammalian target of rapamycin protein (mTOR) pathway that controls cell growth, in part by regulating PI3K activity (19, 20). PTEN loss was noted in numerous tumors, including prostate cancers, breast cancers and gliomas (21–23). In recent years, PTEN loss has also been reported in chordomas (24–26).

### Decreased PTEN expression was associated with reduced PFS and inhibit cell proliferation

PTEN loss is associated with cell growth and tumor progression. Park et al. found the mTOR pathway is activated after PTEN deletion in adult retinal ganglion cells, which robustly induces axon regeneration upon optic nerve damage (27). Penninger et al. reported that PTEN deficiency results in increased proliferation of neural stem cells (28). Le and collaborators found PTEN deficiency is not associated with patient survival but associated with protein kinase B (Akt)/mTOR activation, which may lead to tumor progression (29). PTEN disruption was also correlated with elevated Ki-67 proliferation index, which is a well-known biomarker of tumor progression in chordoma (30). Consistent with former studies, in the present study, decreased PTEN expression in SBC was proved to be associated with reduced PFS, and led to increased tumor cell proliferation. These findings indicate decreased levels of PTEN suppress its inhibitory effect on PI3K/AKT/mTOR signaling, which might lead to increased chordoma cell proliferation.

### PTEN in inhibit invasion and migration in SBC

PTEN deficiency is associated with tumor invasion and migration. Studies demonstrated PTEN deletion might lead to metastatic invasive prostate cancer (31, 32). Masahito et al. demonstrated that overexpression of PTEN inhibits cell migration, growth and focal adhesion (33). In our previous report about factors involved in chordoma with bone invasion, PTEN expression was significantly reduced (14). In the present study, consistent results were obtained. PTEN expression was significantly decreased in SBCs with bone invasion, and its knockdown in tumor cells led to elevated proliferation and invasion. Regarding the mechanism of PTEN in inhibiting tumor invasion and migration, different explanations have been provided. Raftopoulou et al. demonstrated PTEN suppresses cell migration and invasion through its C2 domain but not the PI3K pathway, with the C2 domain activated by phosphorylation at its Thr383 site (18). Wang and collaborators found PI3K/AKT signaling is activated in PTEN-reduced breast cancer with axillary lymph node metastasis. These researchers proposed PI3k/AKT pathway induction is involved in tumor invasion and migration (34). Puzio-Kuter et al. reported mTOR signaling has an important function in prostate cancer invasiveness (32). Thus, this issue requires further investigation.

### Decreased PTEN expression was associated with hard tumor texture

Although the relationship between PTEN expression and tumor texture is rarely described in the literature, PTEN expression was associated with tumor texture in SBCs. PTEN expression level in soft SBCs was increased compared with hard tumors. We learn from Table 3 that the tumor progression rates between soft and hard SBCs were potentially significant (*p* = 0.054, hazard ratio: 1.712, 95% confidence level: 0.974–3.008). Specifically, hard tumors exhibited higher progression rates than soft tumors, indicating that hard tumors grow faster than soft tumors. This difference may be caused by reduced PTEN expression in tumors with hard components. Another explanation is that tumors with reduced PTEN expression are more invasive to surrounding bone, and the bone component makes the tumors harder compared with soft tumors with increased PTEN expression.

The present study included 96 SBC cases with 86 frozen and 65 paraffin-embedded specimens, with available clinical information. the relationship between PTEN and patients' prognosis and clinical characteristics were investigated at protein and gene level respectively, the function of PTEN in chordoma at the cellular level was further verified. This is the largest study to date about PTEN in skull base chordoma, and this is also the first study to demonstrate the role of PTEN in skull base chordoma at protein, genet, and cellular levels. The results indicated that PTEN in SBCs control tumor invasion and tumor progression. These findings provide novel insights into the development of tools for SBC treatment.

Nevertheless, this study had shortcomings. Firstly, not all the patients included contributed both frozen and paraffin-embedded specimens. Secondly, the present study was mainly exploratory, and the mechanism was not adequately studied. Additionally, only one cell line was used *in vitro* experiments caused by the slow growth of other chordoma cell lines, we will repeat the experiment involved in this study in other cell lines in the future.

## Conclusion

PTEN expression in SBCs was assessed at the protein and mRNA levels. In addition, the associations of PTEN expression with patient features were analyzed, and its functions and preliminary mechanism were further verified at the cellular level. The results indicated that PTEN protein expression in SBC was correlated with tumor invasiveness and tumor texture, and decreased PTEN protein and gene expression levels in SBC were associated with reduced PFS. Finally, knockdown of PTEN in tumor cells led to higher proliferation and invasion.

## Contribution to the field statement

Skull base chordoma represents a rarely diagnosed low-grade malignancy. With invasive growth, this tumor damages the surrounding bone seriously and is prone to recurrence after surgery; its long-term prognosis is poor, and there is no effective chemotherapy. Relevant factors and mechanisms involved in skull base chordoma are urgently needed. This study focused on PTEN expression in this tumor. In this study, 96 patients with skull base chordoma were included, including 65 with paraffin-embedded specimens and 86 with frozen specimens. Immunohistochemical staining and qRT-PCR were performed to detect PTEN expression at the protein and mRNA levels, respectively. PTEN expression and the clinical characteristics of SBC patients were next analyzed. Further, the PTEN gene in the chordoma cell line UCH-1 was knocked down using an siRNA, and cell function changes after knockdown were analyzed. Finally, our results revealed decreased PTEN expression in SBC was associated with shorter progression free survival, and PTEN silencing in chordoma cells led to higher cell proliferation and invasiveness.

## Data Availability

The original contributions presented in the study are included in the article/Supplementary Material, further inquiries can be directed to the corresponding author/s.
